# Pulmonary bleeding in racehorses: A gross, histologic, and ultrastructural comparison of exercise-induced pulmonary hemorrhage and exercise-associated fatal pulmonary hemorrhage

**DOI:** 10.1177/03009858221117859

**Published:** 2022-08-16

**Authors:** Guido Rocchigiani, Ranieri Verin, Francisco A. Uzal, Ellen R. Singer, Paola Pregel, Lorenzo Ressel, Emanuele Ricci

**Affiliations:** 1 University of Liverpool, Neston, UK; 2 University of Padua, Padua, Italy; 3 University of California, Davis, Davis, CA; 4 E Singer Equine Orthopaedics and Surgery, Parkgate, UK; 5 University of Turin, Torino, Italy

**Keywords:** exercise-induced pulmonary hemorrhage, hemosiderin, horse, iron encrustation, lung, sudden death, vascular remodeling

## Abstract

Exercise-induced pulmonary hemorrhage (EIPH) is a common condition of Thoroughbred racehorses that is usually responsible for reduced performance, while exercise-associated fatal pulmonary hemorrhage (EAFPH) is characterized by severe pulmonary bleeding of unknown pathogenesis resulting in sudden death during strenuous exercise. The aim of the study was to characterize and compare anamnestic data together with pulmonary gross, histologic, and ultrastructural findings in racehorses with EIPH (n = 10), EAFPH (n = 10), and control horses (n = 5). No differences in anamnesis were identified between the 3 groups. Grossly cranial lobe reddening and edema scores were significantly more prevalent and severe in the EAFPH group compared with the EIPH and control groups. Histologically, hemorrhage scores were higher in the EAFPH group, while hemosiderophages, iron encrustations of collagen and elastin fibers, and vascular remodeling scores were significantly higher in EIPH group compared with the EAFPH and control groups. In all groups, caudal lung locations exhibited a significantly higher score for vascular remodeling, hemosiderophage accumulation, iron encrustation, and type II pneumocyte hyperplasia when compared with cranial, dorsal, and ventral locations. Ultrastructural analysis of perivascular collagen showed fibrils with significantly larger diameters in the EAFPH group compared with the EIPH group but not compared with the control group. This study demonstrates that lungs of horses that experienced EAFPH show significantly less vascular remodeling and other long-term pulmonary abnormalities that characterize horses with EIPH.

Pulmonary hemorrhage is a common clinical condition and *postmortem* finding in equine athletes. The term “exercise-induced pulmonary hemorrhage” (EIPH) was coined by Pascoe et al^15^ in 1981 to describe epistaxis of pulmonary origin, especially after exercise. Since the original paper, many studies have characterized EIPH in flat or jump racehorses, as well as in horses participating in other sports, such as barrel racing and endurance.^9,19^ Exercise-induced pulmonary hemorrhage is believed to be an important cause of reduced athletic performance, especially in cases with severe bleeding.^17^

Typical *postmortem* findings in horses with EIPH are bilateral dark blue to light brown discoloration of the caudo-dorsal pleura and lung. These discolored areas are microscopically characterized by variable accumulation of hemosiderophages and fibrosis of multiple pulmonary microcompartments, including the interstitium, the pleura, and small (100–200 µm in diameter) intralobular veins, the latter known as vascular remodeling (VR).^3^ More specifically, VR is characterized by transmural accumulation of collagen with occasional narrowing of the lumen. In a study on Thoroughbred (TB) horses with EIPH, the distribution of VR in small intralobular veins was most prevalent in the caudo-dorsal pulmonary locations, where EIPH lesions were more frequent.^22^ In addition, accumulation of hemosiderophages and interstitial fibrosis almost never occurred in the absence of VR. All these histological findings suggested a central role for the VR of small intralobular veins in the pathogenesis of EIPH. It was hypothesized that the concentric rings of collagen-affecting small intralobular pulmonary veins (i.e. VR) developed in response to the high blood pressure during exercise, leading to reduced vascular compliance and increased blood pressure in the capillaries, with consequent capillary breakdown.^21^ This hypothesis seems to be reinforced by the co-localization of VR in the same locations where the blood is redistributed during exercise (i.e. caudo-dorsal location).^22^ Other histologic features reported less frequently are bronchiolar distortion, eosinophilic infiltration, basophilia, and Perl’s Prussian blue positivity of collagen and elastic fibers and interstitial edema.^13,21,22^ Ultrastructurally, extravasated erythrocytes and edema within alveolar wall, and gaps between type I pneumocytes and endothelial cells, with basal membrane preservation, were observed.^20^

Exercise-associated fatal pulmonary hemorrhage (EAFPH) is the term first coined in the reference book in 2015 to describe a condition characterized by fatal pulmonary hemorrhages in racehorses and was previously listed amongst the leading causes of sudden death under the term “pulmonary hemorrhages.”^3,10^ Exercise-associated fatal pulmonary hemorrhage is characterized by sudden death during or immediately after the end of exercise.^3^ *Postmortem* features of this condition include widespread bilateral pulmonary edema and hemorrhage, which is more evident in caudo-dorsal locations, accompanied by occasional subpleural pulmonary infarcts and copious blood-tinged froth and frank blood pouring out from both nares when the cadaver is rested on a side. Histologically, EAFPH is characterized by severe hemorrhages involving multiple pulmonary microcompartments, including, but not limited to, alveoli and lobular septa and diffuse pulmonary congestion.^3^ Fatal pulmonary hemorrhage is one of the most frequent causes of sudden death in racehorses, and such lethal pulmonary bleeding has been reported long before the acronym EAFPH was coined.^8,10^ The occurrence of acute cardiac failure or spastic contraction of pulmonary postcapillary sphincters have been listed as possible pathogenetic mechanisms for the occurrence of EAFPH, but this has not been proven.^3^ It is not fully understood whether EAFPH horses show concomitant EIPH lesions (i.e. hemosiderophages accumulation, VR, and fibrosis). The aim of this study was to compare anamnestic data, gross, histologic, and ultrastructural findings of racehorses that died and had lesions compatible with EAFPH, of racehorses that died of causes not related to pulmonary pathology but had incidental EIPH pulmonary lesions (under light microscopy), and of horses that died without pulmonary lesions (control). We hypothesized that racehorses with EAFPH would show significantly less long-term changes and VR than racehorses with EIPH, suggesting, if confirmed, that histopathological lesions of EIPH are not predisposing to fatal pulmonary hemorrhagic events (EAFPH).

## Materials and Methods

All horses included in the study were submitted for postmortem examination to the Laboratory of Veterinary Pathology, University of Liverpool, for diagnostic purposes. Three groups of horses were included in this study. The EIPH group was composed of TB racehorses that were euthanized or died naturally from noncardiopulmonary conditions (e.g. catastrophic fractures) but showed characteristic histologic lesions of EIPH. The microscopic inclusion criterion for this group was the presence of at least 1 cluster of 3 hemosiderophages within the bronchial or bronchiolar lumen in 10 fields of view with a 10× objective (31.4 mm^2^). The EAFPH group was composed of TB racehorses that died during or a few (~ 0–4) hours after a competition with gross and microscopic lesions compatible with EAFPH and in the absence of other potentially fatal lesions. The macro and microscopic criterion for this group was the presence of large volume of uncoagulated blood within the airways and widespread pulmonary hemorrhage confirmed histologically. The control group was composed of horses (TB and other breeds) that were euthanized due to or that died from nonpulmonary-related causes and showed no microscopic lesions compatible with EIPH.

Twenty-five horses were included in the study. Five horses were included in the control group: 2 TBs, 1 Irish draft, 1 Welsh Cob, and 1 Arabian. Exercise-induced pulmonary hemorrhage and EAFPH groups were composed of 10 TB racehorses each.

For each racehorse, anamnestic data including race type (i.e. flat, jump), total number of races run, age, sex, and days passed since last race, were recorded and compared between EIPH and EAFPH groups. Local environmental humidity and temperature at the time of each race were recorded. Race data were retrieved from the Racing Post website (https://www.racingpost.com), while the weather data were retrieved using the closest meteorologic station, on the weather underground website (https://www.wunderground.com).

**Figure 1.**
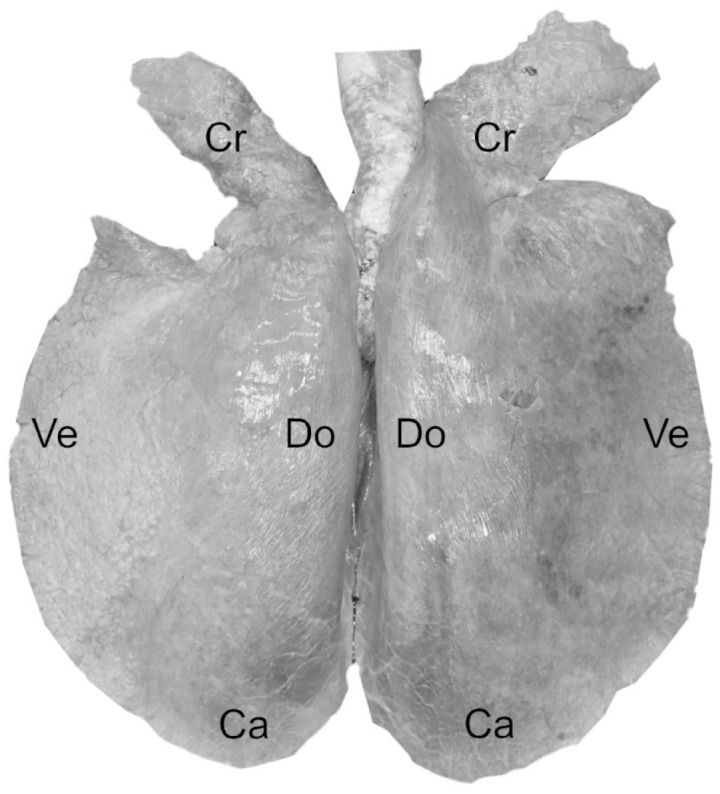
Schematic representation of the lung sampling protocol, illustrating the anatomical locations. Cr, cranial; Ve, ventral; Do, dorsal; Ca, caudal.

Each horse underwent a thorough *postmortem* examination, involving all body systems conducted by GR with other senior board-certified pathologists (LR, ER, and RV). Systematic measurement of the heart/body weight ratio, cardiac ventricular diameters, and wall thicknesses (i.e. left ventricular free wall, interventricular septum and right ventricular free wall) were also included. Measurements were conducted on a transverse section at one-third of the heart height, measured from the cardiac apex to the cardiac base. The larynx was fully evaluated for the presence of potential postsurgical scars, muscular atrophy, and for any other abnormalities. Samples for histology were collected from every horse, and included: brain (frontal cortex, hippocampus, cerebellum, and choroid plexi), stomach (*margo plicatus* area), small intestine, large colon, liver, epiglottis, lungs, heart, ascending aorta, kidney, and spleen. From both lungs, samples of caudal, cranial, dorsal, and ventral locations (Fig. 1) were collected. From the heart, full thickness slices from the right and left ventricular free walls, interventricular septum, and myocardium adjacent to the fibrous trigone (atrio-ventricular node) were collected. All tissue samples were fixed by immersion in 10% formalin, pH 7.4 for at least 48 hours, paraffin embedded, and cut to produce 4-µm-thick sections, before staining them with hematoxylin & eosin (H&E), as per standard protocol. To assess the presence of hemosiderin and VR in the pulmonary sections, Perl’s Prussian blue and picrosirius red staining, respectively, were also performed.

Ultrastructural analysis using transmission electron microscopy was performed on the dorsal location of the left lung of horses from each group, with special emphasis on the alveolar wall and intralobular veins. Cubes of lung tissue (1 mm^3^) were fixed first in 2.5% glutaraldehyde and then in osmium tetroxide 1%, followed by uranyl acetate staining. After dehydration, the sections were embedded in epoxy resin that was polymerized at 60°C overnight. Semithin, 0.5-μm-thick, toluidine blue 1% stained sections were produced to assess target areas for ultrastructural analysis. Ultra-thin sections (75 nm) were then mounted on copper grids and examined under a Philips EM208S (FEI UK, Cambridge, UK) transmission electron microscope (TEM).

For the pulmonary gross pathology findings, a scoring system ranging from 0 (absent) to 3 (severe) was applied to each type of finding, including blue-brown caudo-dorsal discoloration, rib imprints, fibrous tags, pleural hemorrhages, pleural plaques, airway edema and hemorrhage, cranial lobe reddening and edema, and laryngeal hemorrhages. Pleural hemorrhages were defined as raised, red to black, well-demarcated lesions restricted to the pleura. Pleural plaques were defined as raised, well-demarcated, pink to white opaque lesions restricted to the pleura and obscuring the underlying pulmonary parenchyma. Laryngeal hemorrhages included variable degrees of laryngeal reddening and raised mucosal hemorrhages (Supplemental Table S1). Scoring of the gross changes was conducted by one of the authors (GR, blinded to sample identity).

For the pulmonary microscopic findings, a scoring system ranging from 0 (absent) to 3 (severe) was applied to each type of finding (Supplemental Table S2). For each individual pulmonary location (cranial, caudal, ventral, and dorsal locations), the histological scores were recorded as mean score of the left and right lung. For each tissue section, evaluation of the pulmonary parenchyma was performed by analyzing 5 random, nonoverlapping 10× fields (15.7 mm^2^), whereas every portion of interlobular septa and pleura present in the slides was analyzed for these microcompartments. Bronchiolar evaluation was performed by analyzing 5 random bronchioles in each tissue section. Vessels were classified into pleural vessels (vessels within the pleura), pulmonary artery branches (larger vessels adjacent to the bronchioles or bronchi), and intralobular small veins (100- to 200-μm-caliber vessels not adjacent to any septa, pleura, bronchioles, or bronchi) according to their morphology and localization. For each type of vessel, a maximum of10 random nonoverlapping vessels were examined at 10× (31.4 mm^2^). Basophilic discoloration of collagen and elastin coupled with positivity to Perl’s Prussian blue staining was defined as iron encrustation.^18^ Vascular remodeling was defined as increased amount of adventitial collagen around small intralobular pulmonary veins.^22^ Bronchiolar inflammation was evaluated according to the presence of neutrophils, eosinophils, macrophages, lymphocytes, and plasma cells (the latter 2 when not forming lymphoid follicles) around bronchioles (see Supplemental Table 2). An individual score (from 0 to 3, see Supplemental Table S2) for each parameter was calculated for each horse. A combined hemorrhage score was obtained by calculating the mean of the pleural, septal, airway, and alveolar hemorrhage scores for each pulmonary location. A combined hemosiderophage score was obtained calculating the mean of the pleural, septal, peribronchiolar, alveolar, and perivascular hemosiderophage scores for each pulmonary location and vessel type. A combined iron encrustation score was obtained calculating the mean of the pleural, septal, peribronchiolar, alveolar, and perivascular iron encrustation scores for each pulmonary location and vessel type. Vascular remodeling, airway inflammation, and type II pneumocyte hyperplasia scores were evaluated individually (i.e. not combining scores from other micro-compartments) by calculating the mean score of each pulmonary location. Histological scoring was performed by one of the authors (GR, not blinded to sample identity).

Electron microscope images were evaluated qualitatively by searching for ultrastructural changes of alveolar septa (at least 2 areas per horse) and intralobular small veins (at least one vessel per horse). Alveolar septa were evaluated for morphological changes in the endothelial cells, type I, and type II pneumocytes, interstitial fibroblasts, and alveolar lumina. Small intralobular veins were evaluated for morphological changes occurring in the tunica intima, external elastic lamina, tunica muscularis, or tunica adventitia. In addition, cross-sections and longitudinal sections of collagen bundles around small intralobular veins were examined to characterize both fibril diameter and D periodic band length. D periodic band length was calculated as the length of the polar, electron dense, segments visible on longitudinal sections of collagen fibrils. ImageJ software (https://imagej.nih.gov/ij/)^4^ was used to calculate fibril diameter and D periodic band length of 30 representative images of cross-sections and longitudinal sections of fibrils, coming from every horse evaluated with TEM.

GraphPad Prism, version 9.3.0, for Windows (GraphPad Software, San Diego, California USA, www.graphpad.com) was used for statistical analyses. Normality of distributions was verified by means of Kolmogorov and Smirnov tests, and Bartlett’s tests were applied to identify if standard deviations were significantly different among groups. Then, to compare scores obtained in the 3 examined groups, 1-way analysis of variance (ANOVA) followed by Tukey’s multiple comparisons tests was performed for parametric results, whereas, for nonparametric data Kruskal-Wallis tests were applied, followed by Dunn’s multiple comparison tests. Differences were considered significant for *P* < .05. Histological scores were also compared according to their pulmonary locations (i.e. cranial vs. dorsal vs. ventral vs. caudal), regardless of the horses’ groups. For categoric variables (e.g. race type and sex), correlation was assessed using a Pearson’s chi-square test.

## Results

Anamnestic data are reported in Supplemental Table S3 and Fig. S1. Age and sex were not significantly different among all experimental groups. Racing and weather data were not significantly different between EIPH and EAFPH group. Heart/body weight ratios were available for 2, 9, and 8 horses from the control, EIPH, and EAFPH groups, respectively. Heart/body weight ratios were not significantly different among groups and ranged from 0.66 to 0.97 (mean: 0.81), 0.87 to 1.19 (mean: 0.98) and 0.88 to 1.1 (mean: 0.97) in control, EIPH, and EAFPH groups, respectively.

**Figure 2.**
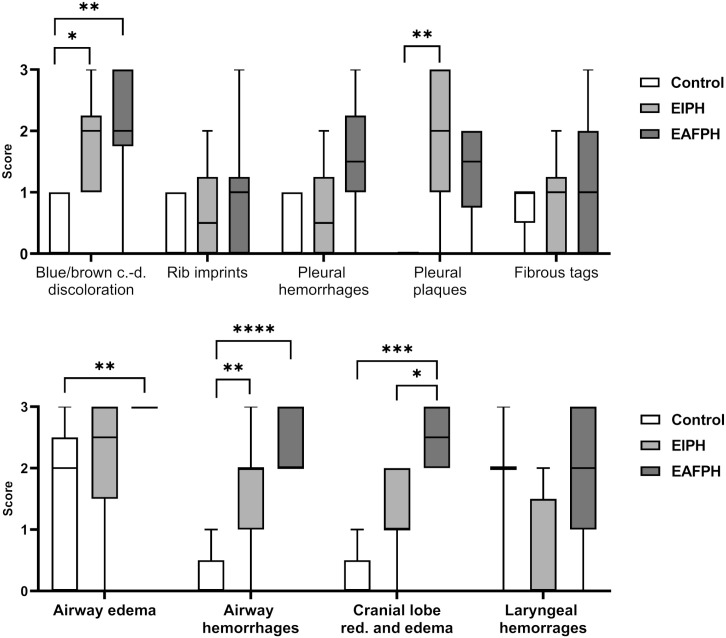
Comparison of macroscopic mean scores between control (N = 5), exercise-induced pulmonary hemorrhage (EIPH) (N = 10), and exercise-associated fatal pulmonary hemorrhage (EAFPH) (N = 10) horses. EIPH and EAFPH horses showed higher scores for blue/brown discolouration, airway hemorrhages, pleural plaques, and cranial lobe reddening and edema than control horses. EAFPH horse showed higher cranial lobe reddening and edema score compared to EIPH horses. c.-d., caudo-dorsal; red., reddening. *P < .05. **P < .01. ***P < .001. ****P < .0001.

Dark blue-light brown caudo-dorsal discoloration and airway hemorrhage scores were significantly higher in EIPH and EAFPH groups compared to the control group (*P* = .0049 and *P* < .0001, respectively). Histologically, pleural plaques were characterized by accumulation of collagen expanding the pleura and variable numbers of newly formed vessels, hemorrhage, hemosiderophage, and spindle cells (likely myofibroblasts). Pleural plaques were present in 0/5, 9/10, and 8/10 horses from the control, EIPH, and EAFPH groups, respectively. Pleural plaque scores were significantly higher in EIPH horses compared to the control group (*P* = .0049). Airway edema was significantly greater in the EAFPH group compared to control group (*P* = .0069), but not in comparison to the EIPH group. Cranial lobe reddening and edema scores were significantly different among all 3 groups (*P* = .0002), with the control group showing the lowest and the EAFPH group the highest scores. Laryngeal evaluation did not detect postsurgical scars or muscular atrophy in any horse. The other macroscopic finding scores were not significantly different among groups (Fig. 2).

**Figure 3.**
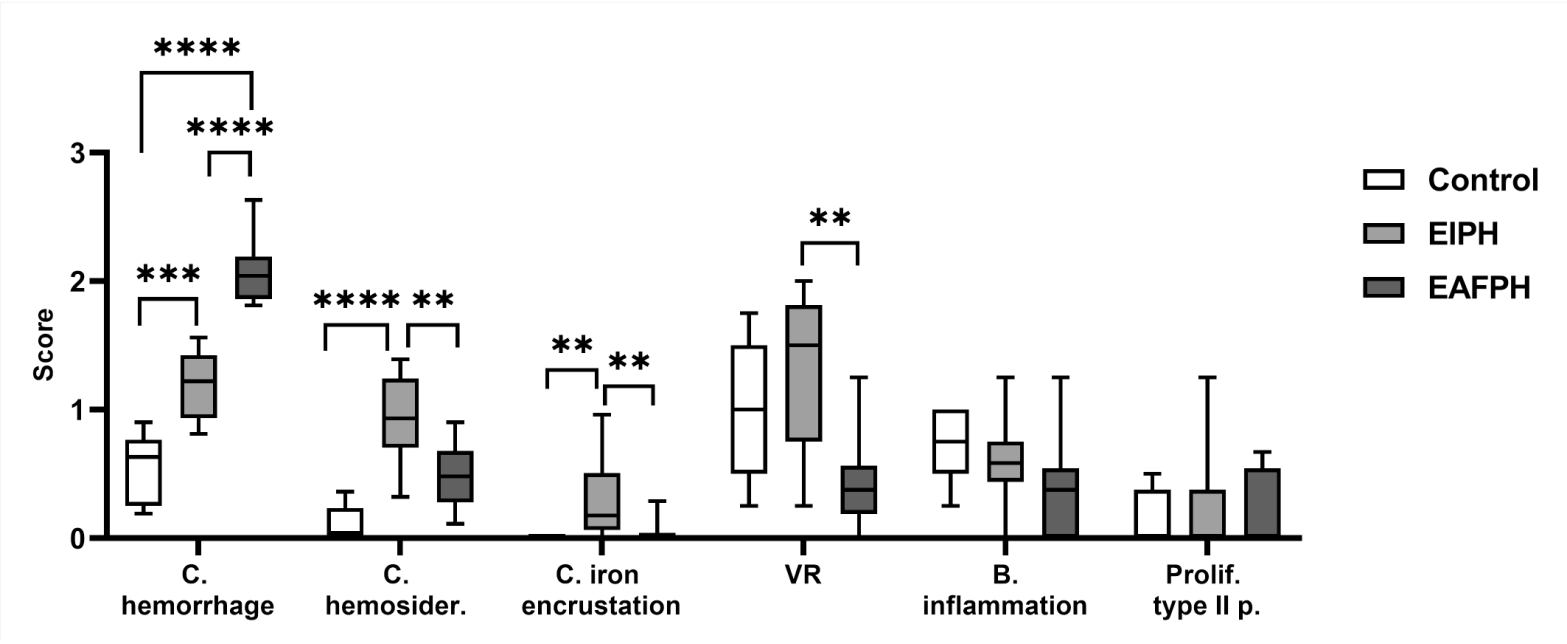
Comparison of microscopic mean scores between control (N = 5), exercise-induced pulmonary hemorrhage (EIPH) (N = 10) and exercise-associated fatal pulmonary hemorrhage (EAFPH) (N = 10) groups. EIPH horses exhibited higher scores of hemosiderophages, iron encrustation and VR compared to EAFPH horses. EAFPH showed higher hemorrhage score compared to all other groups. C., combined; hemosider., hemosiderin; VR, vascular remodeling; B., bronchiolar; Prolif., proliferation; p., pneumocyte. **P < .01. ***P < .001. ****P < .0001.

Regarding the histopathological examination, combined hemorrhage scores were significantly different among all groups (*P* < .0001), with the control group exhibiting the lowest and the EAFPH group the highest scores (Fig. 3). A similar trend was observed in all microcompartments (Supplemental Fig. S2). Combined hemosiderophage scores were significantly higher in the EIPH group compared to the other 2 groups (*P* < .0001; Fig. 3). Aggregates of alveolar hemosiderophages (i.e. at least one) within the alveolar lumina were observed in 3/5, 10/10, and 10/10 horses from the control, EIPH, and EAFPH groups, respectively. In the control group, hemosiderophages were more frequently observed in alveolar lumina, while hemosiderophages were fewer to absent in the pleura, septa, peribronchiolar collagen, and vessel adventitia. In the EIPH and EAFPH groups, hemosiderophages were more frequently encountered in all the microcompartments examined compared to control groups, although not always simultaneously. Hemosiderophages in EIPH horses were also more frequently observed within the pulmonary arteries and intralobular small veins adventitia than in EAFPH horses (Supplemental Fig. S3). Iron encrustation was diffusely Perl’s Prussian blue positive and Von Kossa negative (data not shown). Iron encrustation of, at least, a single microcompartment (e.g. pleura or interstitium) was present in 0/5, 9/10, and 3/10 horses from the control, EIPH, and EAFPH groups, respectively. Combined iron encrustation scores were significantly higher in EIPH group compared to EAFPH and control groups (*P* = .0011), with no statistical difference between control and EAFPH groups. Iron encrustation of intralobular small veins was more frequently observed in EIPH horses compared to all other groups (Supplemental Fig. S4). Vascular remodeling was significantly higher in the EIPH compared to the EAFPH group (*P* = .002) but not compared to the control group. Type II pneumocyte hyperplasia and bronchiolar inflammation were not significantly different among the groups (Fig. 3).

Histopathological scores were compared between pulmonary locations (caudal, ventral, cranial, and dorsal) for all groups in combination. Total hemosiderophage scores were significantly higher in the caudal location compared to all other locations (*P* < .001), while the dorsal location also showed significantly higher scores compared to the ventral and cranial locations. Total iron encrustation scores were significantly higher in the caudal location compared to the other 3 locations (*P* = .001), while no iron encrustation was detected in any microcompartment of the cranial lobes. Vascular remodeling was significantly higher in the caudal location if compared to cranial and ventral regions (*P* = .006) but not if compared to the dorsal location. Type II pneumocyte hyperplasia scores were significantly higher in the caudal region than in all other locations (*P* = .007; data not shown).

Ultrastructural analysis was conducted on 2 control, 3 EIPH, and 3 EAFPH horses. In the perivascular (adventitial) collagen of intralobular small veins, collagen fibril diameters were significantly larger in the EAFPH group compared to the EIPH group (*P* = .03), with the EAFPH group showing also increased variance (133.9 compared to 54.1 nanometers, respectively) but not compared to controls; no significant differences regarding the length of D periodic bands were observed (Supplemental Fig. S5). Small intralobular veins and interalveolar septa did not show any other morphological differences among groups.

Gross and histopathological analysis of the other organs revealed occasional abnormalities of incidental nature or negligible relevance in the pathophysiology of the pulmonary changes at the core of this study (e.g. distal limb fracture and intestinal torsion with infarction).

## Discussion

This study characterized and compared the gross, histopathological, and ultrastructural pulmonary changes of racehorses that died during or soon after racing competitions with lesions consistent with either EIPH or EAFPH. We documented distinctive changes that help to differentiate the 2 conditions, providing insight into their characteristic pathologic features.

Our findings support the initial hypothesis that EAFPH horses show significantly less VR (of small intralobular pulmonary veins) than EIPH horses, which is considered hallmark for EIPH pathogenesis.^21^ The absence of severe and frequent VR in most of EAFPH horses suggests that EAPFH does not need long-standing EIPH to take place. This finding suggests that the severe pulmonary hemorrhage observed in EAFPH racehorse does not represent the long-term exacerbation of EIPH, but a more extensive and acute process; furthermore, this finding might also suggest that EIPH horses are not at increased risk to develop EAFPH. Considering the relative “nonlethal” role of EIPH in equine pathology (supported by our findings), it seems even more fundamental to distinguish EIPH from the EAFPH, even if they both manifest as pulmonary hemorrhages.

Our study also reveals that VR, which was originally hypothesized to be responsible of EIPH,^21^ was also noted with relative frequency in control horses, challenging the view of a crucial role for VR in the pathogenesis of EIPH. The control group exhibited surprisingly high scores for intralobular VR with 3 out of 5 horses exhibiting intralobular small veins with VR score >1 (Fig. 3). Except for one control TB racehorse, the other horses were an Irish draught cross and a Welsh horse who died due to a gastric rupture and a *Strongylus vulgaris*–associated aortic rupture, respectively. The VR in these control horses showed morphological similarity (i.e. dense collagen within intralobular vein adventitia) with previously reported grade II VR.^22^ Further studies are needed to properly characterize the VR and pathological relevance in non-TB horses. Thus, in this study, VR of small intralobular pulmonary veins has been detected in control group animals, indicating that such a lesion is not specific to EIPH or exclusive to racehorses.

Another commonly observed histological change in EIPH and in some EAFPH horses was iron encrustation. This basophilic discoloration affected pleural, adventitial, peribronchiolar and interalveolar collagen and elastin, and had a similar appearance to dystrophic mineralization observed in routine diagnostic pathology. Nevertheless, Perl’s Prussian blue positivity and the frequent association with surrounding hemosiderophages seems suggestive of a pathogenesis most likely linked to the breakdown pathway of the hemoglobin or at least indicating that iron (confirmed with Perl’s staining, data not shown) constitute such basophilic encrustations. The regional distribution of iron encrustation mirrored the distribution of hemosiderophages, showing the highest score in the caudal lungs, where VR was also most severe. Although the exact pathogenesis by which recurrent hemorrhages cause basophilic discoloration of collagen and elastin in the lung is poorly understood,^1^ a similar histological appearance is reported in people with pulmonary veno-occlusive disease.^18^ In veterinary literature, iron encrustation is described in one case report in a cat,^23^ while in horses, iron encrustation is cited in few EIPH studies, without attributing any particular importance to its presence.^13,21^ Since we found iron encrustation in at least one microcompartment in the majority of EIPH horses (9/10), with none present in the control group, we propose that iron encrustation should be included in the list of the EIPH lesions, together with VR, fibrosis, and hemosiderophage accumulation. Iron encrustation of at least one microcompartment was observed only in 3 EAFPH horses and with lower intensity compared to the EIPH horses, a finding which might be useful to distinguish these 2 entities.

The significantly higher score for total hemosiderophages in the EIPH group was expected because EIPH manifests commonly with hemosiderophage accumulation across multiple microcompartments. Nevertheless, small numbers of alveolar hemosiderophages were present in all EIPH and EAFPH horses investigated and, occasionally in some control group horses (3/5). This finding seems in agreement with previous clinical studies, in which almost all TB horses in training showed numerous hemosiderophages in the bronchoalveolar lavage cytology.^11^ These findings suggest that a small number of hemosiderophages are commonly present in equine alveolar lumina and the diagnosis of EIPH should not solely rely on their presence/absence but on their relative proportion.

Our data support the widespread evidence that hemosiderophages are more consistently present in the caudal and dorsal locations compared to cranial and ventral ones, irrespective of the group to which the horse belonged. In terms of microcompartment distribution, our results highlight that few differences were present between EIPH and EAFPH horses, with higher amount of hemosiderophages in the adventitia of pulmonary arteries and intralobular small veins in the EIPH group but not in the pleural vessels (Supplemental Fig. S3); such finding can be interpreted as a result of a blood flow alteration present in the pulmonary circulation (i.e. pulmonary arteries and small intralobular pulmonary veins) but absent in the systemic circulation (as pleural vessels are served from the systemic circulation).

Another interesting histological finding was the presence of type II pneumocyte hyperplasia in multiple TB racehorses. The type II pneumocyte hyperplasia was randomly distributed but with higher predilection for the subpleural alveoli, where hemosiderophages, pleural hemorrhages, and fibrosis (pleural plaques) also colocalize. This finding led to the hypothesis that type II pneumocyte hyperplasia, a widely accepted expression of alveolar repair after mechanical damage,^6,14^ occurs in areas exposed to greater mechanical tension and stretching determined by high alveolar transmural pressure following alveolar over-distension during athletic peak performance.

Regarding the macroscopic findings, the gross lesion that significantly diverged between EIPH and EAFPH groups was the cranial lobe reddening and edema. According to our results, bilateral reddening of the cranial lobes indicates that hemorrhagic foci and interstitial edema consistently affected the cranial lobes and that such a change is highly characteristic of EAFPH. This finding is in accordance with the literature where lesions of EIPH are mostly restricted to the caudo-dorsal lungs, rarely extending to the cranial lobes,^12^ unlike EAFPH.

Examination of pleural plaques revealed that they were absent in the control group; supporting the hypothesis that such lesions, which are extremely common in racehorses (i.e. 17/21 racehorses exhibited these lesions), are likely linked to the racing activity, and remain uncommon in nonracing horses (unpublished observation of GR and ER). Pleural plaques are likely the result of localized remodeling of previous acute subpleural bleeding because the plaques are often intermixed with areas of acute and subacute pleural hemorrhages and are more prevalent and more obvious in EIPH horses. Furthermore, pleural plaques are co-localized with underlying parenchymal changes such as acute and long-term alveolar hemorrhage and type II pneumocyte hyperplasia. Thus, it is possible that the aforementioned cohort of pathological changes (pleural hemorrhages and plaques, and type II pneumocyte hyperplasia) share a similar pathogenetic mechanism, which appears to induce alveolar and pleural damage contemporaneously in the same area.

Laryngeal evaluation did not reveal any lesions that would predispose to a possible static occlusion of the larynx. It has been suggested that any type of obstruction in the upper airways could be a contributing factor in the pathogenesis of EIPH and sudden death.^5^ Our findings present no evidence to support laryngeal static obstruction as cause of neither EIPH nor EAFPH, such as evidence of postoperative laryngeal scars or atrophy of cricoarytenoid muscles. The only changes affecting the larynx in our experimental groups of horses are ascribable to the cranial “reflux” of abundant red froth and frank blood associated with intense mucosal hemorrhages.

All other macroscopic changes appeared as poor discriminators for distinguishing EIPH from EAFPH. What is surprising is that even horses that died or were euthanized for fractures can show moderate volume of uncoagulated blood in the airways; therefore, any pathologist should resist the temptation of making an EAFPH diagnosis when a small to moderate volume of blood is present within the trachea/major bronchi, unless diffuse acute alveolar hemorrhages are confirmed by histopathology and/or profuse bleeding fills the upper airways draining from markedly hemorrhagic lungs.

The TEM analysis, which focused on the perivascular adventitial collagen, revealed no differences in the length of the D-period, whereas significant differences were observed regarding the diameter of collagen fibrils that were significantly smaller in diameter in EIPH horses when compared to EAFPH horses. This finding has to be analyzed with consideration that newly formed collagen, in the context of repair and regeneration, is first achieved by deposition of smaller diameter fibrils which, subsequently are remodeled to “normal size.”^2^ Despite the reduced statistical power of such an observation, due to smaller sample size and variability in specimen orientation and preservation, collagen fibrils of smaller diameter in EIPH may be due to regeneration, in comparison to the larger diameter fibrils in EAFPH that have significantly less long-term alterations in these vessels. Interestingly, collagen fibril diameter did not differ between control and EAFPH horses, possibly suggesting that control horses were likely undergoing active regeneration of collagen fibers of intralobular veins; alternatively, it is also possible that EAFPH and control horses showed “normal” perivascular collagen, unlike EIPH. To further complicate the scenario, collagen fibril diameter can be regulated by a plethora of other molecules, including small leucine-rich proteoglycans, and fibril diameter can vary with aging, which was not evaluated in this study.^16^ Understanding the exact cause of collagen fibril diameter variation in the horses in this study and the role of fibril diameter in vascular pathology remains to be elucidated.

The main limit of this study is the small number of horses evaluated. Moreover, some sampling was limited due to the degree of autolysis, which rendered some tissues unsuitable for inclusion in the study. In particular, autolysis was consistently more advanced in EAFPH horses than in EIPH horses (data not shown). Possibly, despite an equal or even shorter *postmortem* interval, the high body temperature of the horses who died suddenly at the peak of an exhausting athletic performance, coupled with the large volume of extravasated blood within the alveoli, could have provided favorable ground for the quicker onset and progression of autolysis in EAFPH horses.

**Figure 4.**
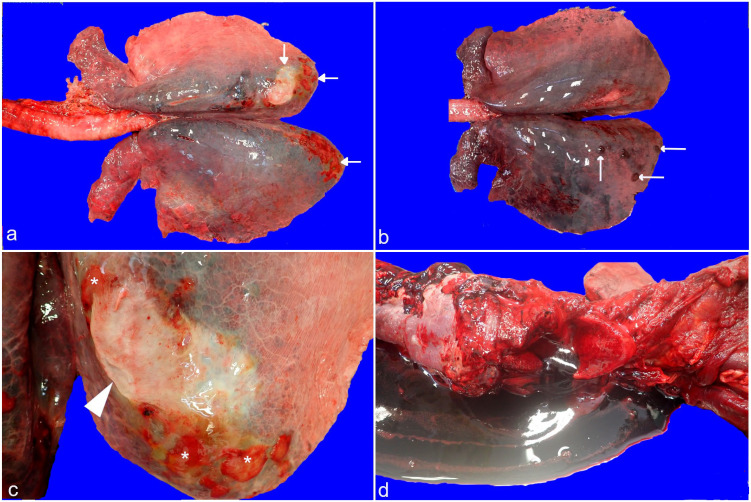
Exercise-induced pulmonary hemorrhage (EIPH) and exercise-associated fatal pulmonary hemorrhage (EAFPH), macroscopic changes in racehorse respiratory system: (a) Case 10, lung, EIPH, bilateral dark blue dorso-caudal discolored areas, with multifocal to coalescing red and white pleural plaques (arrows) on both caudal locations. Cranial lobes are within normal limits. (b) Case 22, lungs, EAFPH, widespread dark red discoloration extending bilaterally from the caudal lung tip to the cranial and lateral regions. Multiple raised dark red elements suggestive of infarcts (arrows). The cranial lobes show bilateral reddening and interstitial edema. (c) Case 10, lung, EIPH, closer view of the pleural surface with a focal white pleural plaque (arrowhead), together with multiple pale red smaller plaques (asterisks). (d) Case 21, larynx and trachea, EAFPH. Large volume of uncoagulated blood draining from the lung after gentle laryngeal handling.

**Figure 5.**
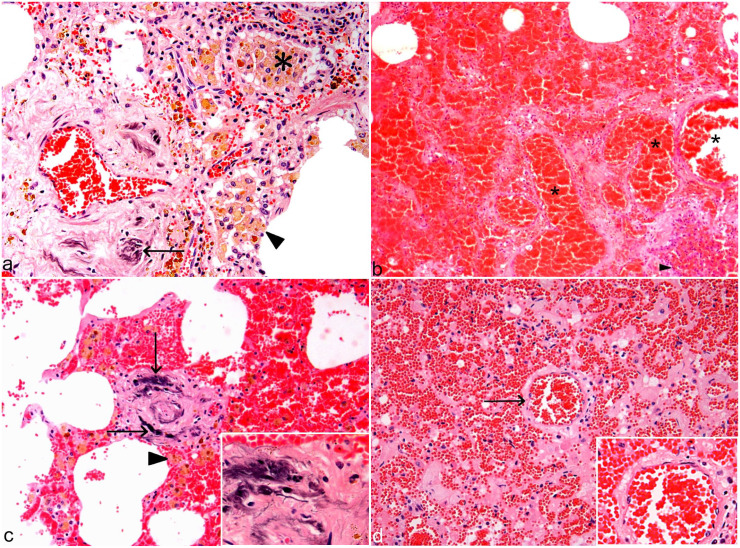
Exercise-induced pulmonary hemorrhage (EIPH) and exercise-associated fatal pulmonary hemorrhage (EAFPH), microscopic pulmonary changes in racehorses: (a) Case 7, EIPH, multiple hemosiderophages within the bronchiolar lumen (asterisk), as well as alveolar lumina (arrowhead) with perivascular iron encrustation (arrow). Hematoxylin and eosin (HE). (b) Case 16, EAFPH, diffuse alveolar hemorrhage expanding all the alveolar spaces with markedly congested vasculature (asterisks) and hemorrhage within bronchiole (arrowhead). HE. (c) Case 9, EIPH, small intralobular vein with severe circumferential intramural collagen deposition which narrows the lumen together with multifocal basophilic discoloration of the collagen (i.e. iron encrustation—arrows); alveolar hemorrhage is surrounding rare hemosiderophages (arrowhead). Inset: higher magnification of the remodeled vessel wall. HE. (d) Case 24, EAFPH: Small intralobular vein within normal thickness (arrow) and with surrounding congestion and hemorrhage. Inset: higher magnification of the within normal limits vessel wall. HE.

Comparing EIPH and EAFPH in TB racehorses, EIPH horses are dominated by diffuse long-term changes, encompassing increased number of hemosiderophages, iron encrustation, and VR, mostly in the caudal and dorsal locations (Figs. 4 and 5). On the contrary, EAFPH horses were characterized by acute abnormalities, such as widespread and severe hemorrhages, which were evident throughout all lung locations, including the cranial lobes (Figs. 4 and 5).

In conclusion, this article highlights several features of EIPH and EAFPH in relation to both anamnestic data, gross, histological, and ultrastructural morphology, paving the way for novel pathogenetic theories. Since the triad of obvious histopathological changes that characterized EIPH (VR, iron encrustation and hemosiderophages) are rare or absent in EAFPH, the results of this study indicate that advanced EIPH lesions do not predispose the racehorse to EAFPH, in line with ACVIM (American College of veterinary Internal Medicine) EIPH consensus statement, in which EIPH is not considered a predisposing factor to other pulmonary diseases.^9^ Whereas the cohort of histopathological alterations observed in EIPH horses is interpreted as the consequence of repeated previous episodes of bleeding with tissue organization, the conspicuous alveolar hemorrhages of EAFPH are the morphological expression of an acute process, differentiated from EIPH by clinicopathological severity, chronicity, and extent of tissue involvement. This study shows significant divergence in the lesions of these hemorrhagic pulmonary syndromes, suggesting potentially different pathogeneses. In other words, it is possible that the 2 conditions (EIPH and EAFPH) are clinicopathological manifestations of the same pathogenetic mechanism leading to either a long-term and mild or acute and fatal pulmonary bleeding or the morphological expression of 2 separated pathogenetic mechanisms, whose identification remains elusive.

## Supplemental Material

sj-pdf-2-vet-10.1177_03009858221117859 – Supplemental material for Pulmonary bleeding in racehorses: A gross, histologic, and ultrastructural comparison of exercise-induced pulmonary hemorrhage and exercise-associated fatal pulmonary hemorrhage Click here for additional data file. Supplemental material, sj-pdf-2-vet-10.1177_03009858221117859 for Pulmonary bleeding in racehorses: A gross, histologic, and ultrastructural comparison of exercise-induced pulmonary hemorrhage and exercise-associated fatal pulmonary hemorrhage by Guido Rocchigiani, Ranieri Verin, Francisco A. Uzal, Ellen R. Singer, Paola Pregel, Lorenzo Ressel and Emanuele Ricci in Veterinary Pathology

sj-xlsx-1-vet-10.1177_03009858221117859 – Supplemental material for Pulmonary bleeding in racehorses: A gross, histologic, and ultrastructural comparison of exercise-induced pulmonary hemorrhage and exercise-associated fatal pulmonary hemorrhage Click here for additional data file. Supplemental material, sj-xlsx-1-vet-10.1177_03009858221117859 for Pulmonary bleeding in racehorses: A gross, histologic, and ultrastructural comparison of exercise-induced pulmonary hemorrhage and exercise-associated fatal pulmonary hemorrhage by Guido Rocchigiani, Ranieri Verin, Francisco A. Uzal, Ellen R. Singer, Paola Pregel, Lorenzo Ressel and Emanuele Ricci in Veterinary Pathology
