# Supplementary material for: Pulmonary bleeding in racehorses: A gross, histologic, and ultrastructural comparison of exercise-induced pulmonary hemorrhage and exercise-associated fatal pulmonary hemorrhage
Source: Vet Pathol. 2022 Aug 16;59(6):973–82. doi: 10.1177/03009858221117859 (PMC9530536; doi:10.1177/03009858221117859)
Supplement: sj-pdf-2-vet-10.1177_03009858221117859 – Supplemental material for Pulmonary bleeding in racehorses: A gross, histologic, and ultrastructural comparison of exercise-induced pulmonary hemorrhage and exercise-associated fatal pulmonary hemorrhage [file sj-pdf-2-vet-10.1177_03009858221117859.pdf]

Supplementals, Rocchigiani et al. Pulmonary bleeding in racehorses

Supplemental figure S1. Comparison of race data between exercise-induced pulmonary hemorrhage (EIPH) (N = 10) and exercise associated fatal pulmonary hemorrhage (EAFPH) (N = 10) racehorses. a: number of total starts. b: days passed since last race. No significant difference observed.

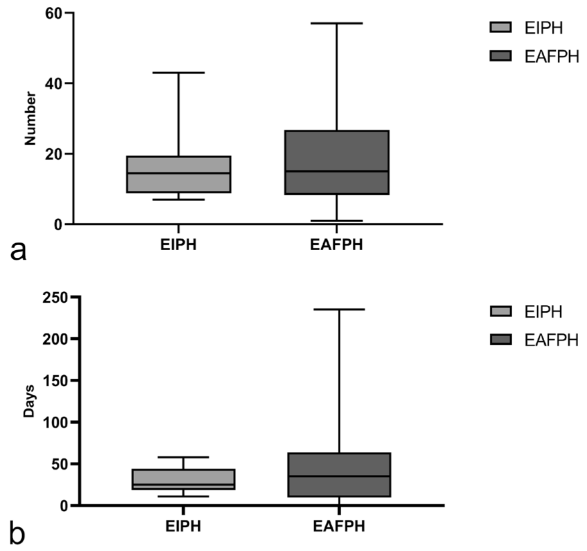

Supplemental figure S2. Histological hemorrhage mean scores divided by micro-compartment and group. Control (N = 5), EIPH (N = 10), and EAFPH (N = 10) horses. I = interlobular; parench. = parenchyma. * = P< 0.05; ** = P< 0.01; *** = P< 0.001; **** = P< 0.0001.

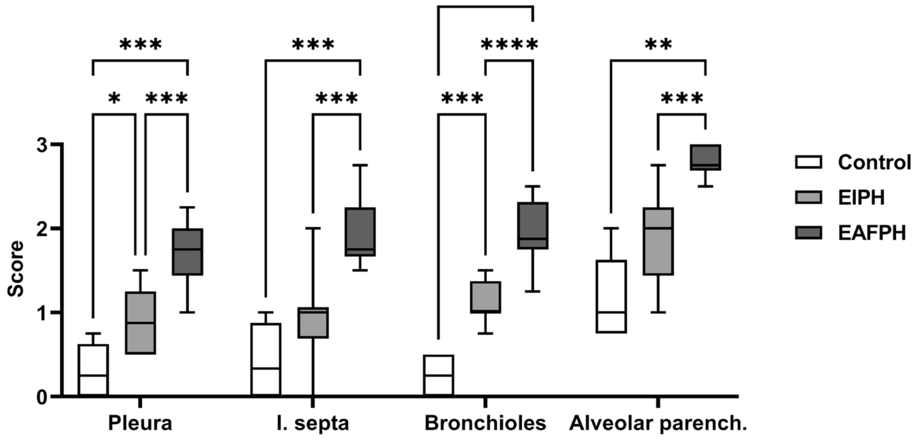

Supplemental figure S3: Histological hemosiderophage mean scores divided by micro-compartment and group. Control (N = 5), EIPH (N = 10), and EAFPH (N = 10) horses. I = interlobular; parench. = parenchyma; Pulm. = pulmonary; Intr. = intralobular * = P< 0.05; ** = P< 0.01; *** = P< 0.001; **** = P< 0.0001.

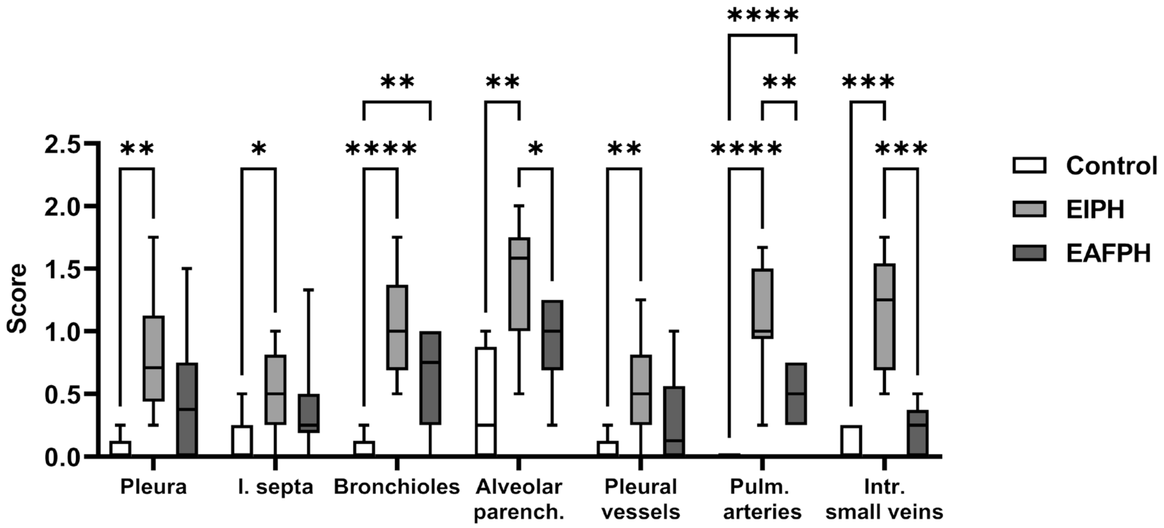

Supplemental figure S4: Histological iron encrustation mean score divided by micro-compartment and group. Control (N = 5), EIPH (N = 10), and EAFPH (N = 10) horses. I = interlobular; parench. = parenchyma; Pulm. = pulmonary; Intr. = intralobular; * = P< 0.05

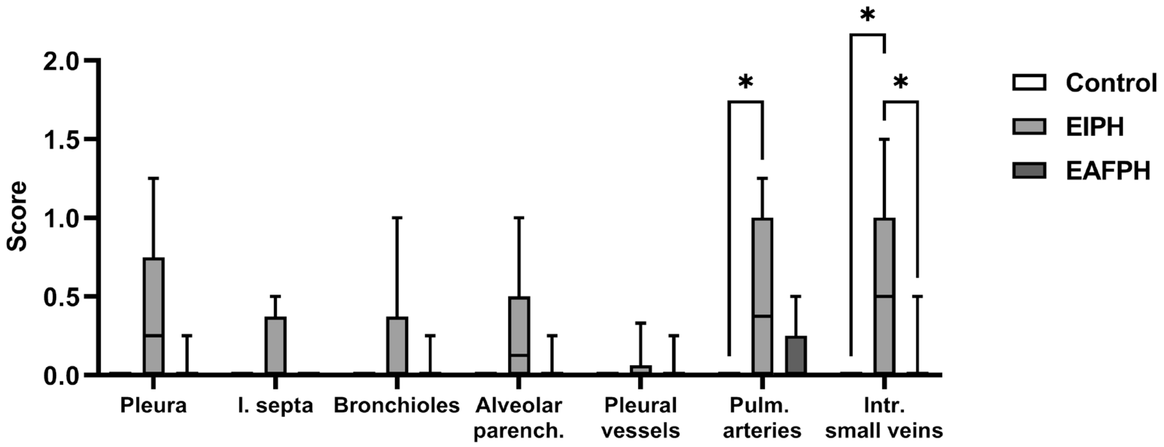

Supplemental figure S5: Transmission electron microscopy (TEM) comparison of perivascular collagen measures of intralobular pulmonary veins between EIPH and EAFPH horses. **a)**. Case 10, cross-sectioned collagen fibrils of an EIPH horse. Bar = 100 nm, TEM. **b)** Case 19, cross-sectioned collagen fibrils of an EAFPH horse. Bar = 100 nm, TEM. **c)** Comparison of collagen fibrils diameter between control (N = 2), EIPH (N = 3) and EAFPH (N = 3) horses. * = P< 0.05. **d)** Comparison of collagen D period length between control (N = 2), EIPH (N = 3) and EAFPH (N = 3) horses.

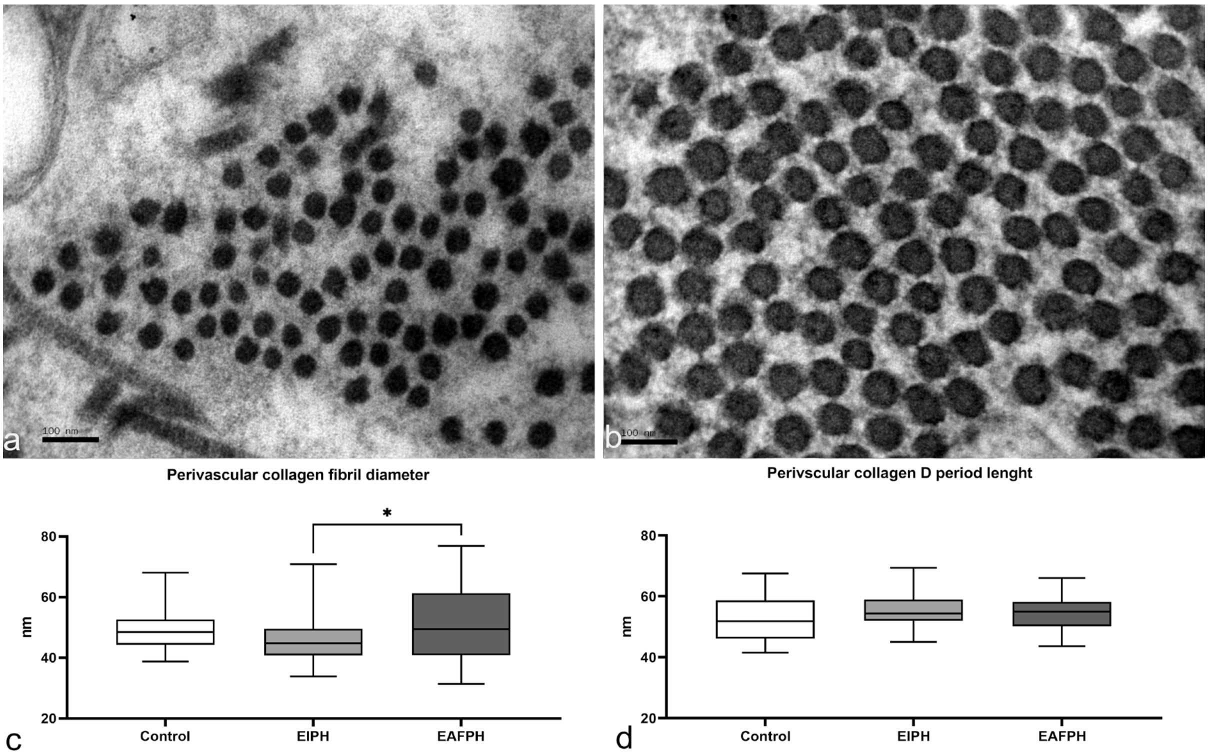

Supplemental table S1: Macroscopic respiratory finding scoring system

	0	1	2	3	Notes
**Blue-brown caudo-dorsal discoloration**	None	Multifocal to coalescing, occurring in one or two lung, restricted to the caudal third of the lungs	Multifocal to coalescing, forming large continuous areas of discoloration extending not exceeding the cranial half of the lungs	Extension exceding the cranial half of the lungs, bilateral and also exteding to the ventral areas	
**Rib imprints**	None	Barely appreciable	Mostly unilateral, visible on the surface of the caudal lung	Bilaterally evident over both lungs, up to the cranial half of the lung	
**Pleural hemorrhages**	None (< 5% of total lung surface)	Mild, mostly scattered and restricted to the lateral margins of one lung (5-20% of total dorsal pleural surface)	Moderate, forming larger coalescing foci (20-40% of total dorsal pleural surface)	Multifocal to coalescing confluent into a single large plaque covering a vast area of one or both lungs (More than 40% of total dorsal pleural surface)	
**Pleural plaques**	None (< 5% of total lung surface)	Mild, mostly scattered and restricted to the lateral margins of one lung (5-20% of total dorsal pleural surface)	Moderate, forming larger coalescing foci (20-40% of total dorsal pleural surface)	Multifocal to coalescing confluent into a single large plaque covering a vast area of one or both lungs (More than 40% of total dorsal pleural surface)	
**Fibrous tags**	None	Rare isolated small tags on margin of a single lung lobe	Multiple, discontinuous segments of the lung ventral margins	Large and continuous areas of ventral margins on both lung showing tags	
**Airway hemorrhages**	None	Small volume of frank blood, not extending past the bronchial bifurcation	Moderate volume of frank blood extending to the trachea	Large volume of frank blood extending to the nostrils	In case of concomitant pulmonary edema, score is 1, 2, and 3 if the edema is pink, red, or dark red, respectively
**Airway edema**	None	Small volume (up to the bronchial bifurcation) of white froth	Moderate volume (up to the larynx) of white to pale red pulmonary edema. A small volume of frank blood is possible	Large volume (up to the nostrils) of white to pale red pulmonary edema.	
**Cranial lobes reddening and edema**	Cranial lobes shows minimal thickened interstitial pattern and unilateral mild reddening	Cranial lobes show mild unilateral, dark reddening or increased interstitial thickening	Cranial lobes display moderate bilateral dark reddening with unilateral dark red raised pleural lesions and/or increased interstitial thickening	Cranial lobes display severe bilateral dark reddening with occasional dark red raised pleural lesions and increased interstitial thickening extending to the cranial part of the caudal lobes	
**Laryngeal hemorrhages**	The larynx shows none or mild congestion (<50% larynx mucosa showing reddening)	The larynx is extensively congested (> 50% larynx mucosa showing reddening)	The larynx is extensively congested and multiple mucosal raised hemorrhagic foci are present	The larynx is diffusely congested and multiple raised haemorrhagic foci forming large coalescing haemorrhagic plaques are present on the mucosa or in the surrounding soft tissue	

Supplemental table S2: Microscopic respiratory finding scoring system

		0	1	2	3	Notes
**Pleura**	Hemorrhage	None	Scattered erythrocytes not forming clusters are in at least 2, 10X HPFs	Moderate hemorrhages forming clusters in 2-3, 10X HPFs	Large hemorrhages expanding the pleura in 4-5, 10X HPFs	
	Hemosiderophages	None	Scattered hemosiderophages not forming clusters are in < 2, 10X HPFs	Moderate number hemosiderophages forming rare clusters in 2-3, 10X HPFs	Numerous hemosiderophages forming common clusters in 4-5, 10X HPFs	
	Iron encrustation	None	Scattered collagen fibers display iron encrustation in < 2, 10X HPFs	Multiple collagen fibers display iron encrustation in 2-4, 10X HPFs	Numerous fibers showing iron encrustation in more than 4, 10x HPFs	
**Septa**	Hemorrhage	None	Scattered isolated erythrocytes are in <2 10 HPFs	Multiple erythrocytes forming clusters within the septa in 2-3, 10X HPFs	Large amount of erythrocytes expanding the septa in 2-3, 10X HPFs	
	Hemosiderophages	None	Scattered hemosiderophages not forming clusters in < 2, 10X HPF	Moderate number of hemosiderophages forming rare clusters in 2-3, 10X HPF within the septal collagen	Numerous hemosiderophages forming common clusters are present in 4-5 10X HPFs.	
	Iron encrustation	None	Scattered collagen fibers display iron encrustation in <10% of the septa	Multiple collagen fibers display iron encrustation in 10-30% of the septa	Numerous collagen fibers display iron encrustation in > 30% of the septa	
**Bronchioles**	Intraluminal hemorrhage	None	Small number of erythrocytes are within the lumen of at least 3 bronchioles	Moderate number of erythrocytes partially occluding the lumen in >3 bronchioles	Large number of erythrocytes occluding the lumen in more than 3 bronchioles	
	Peri-bronchiolar hemosiderophages	None	Scattered hemosiderophages in < 2 bronchioles	Moderate number of hemosiderophages forming rare clusters in 2-3 bronchioles	Numerous hemosiderophages forming clusters in 4-5 bronchioles, occasionally spilling into the lumen	
	Peribronchiolar inflammatory cells (I.C.)	Very rare I.C. are observed in not more than 2 bronchioles	Scattered I.C. forming clusters extending to the submucosa in at least two bronchioles	Moderate number of I.C., forming up to 1 continuous layer within the airway stroma/l.p. in at least two bronchioles	Large number of I..C., forming > 1 layers within airway stroma/l.p. in at least two bronchioles	Focal clusters of lymphocytes and/or plasma cells (BALT) are not considered I.C.
	Peri-bronchiolar iron encrustation	None	Scattered peri-bronchiolar collagen fibers display iron encrustation in 1 bronchiole	Multiple peri-bronchiolar collagen fibers display iron encrustation in 2-3 bronchioles	Numerous peri-bronchiolar collagen fibers display iron encrustation in 4-5 bronchioles	
**Alveolar parenchyma**	Hemorrhage	Present in less than 10% of parenchyma	Present in 10-30% of parenchyma. Usually, composed of sparse, loosely packed erythrocytes	Present in 30-50% of parenchyma. Usually, forming occasional clusters and separated by the septa from empty alveoli	Present in > 50% of parenchyma, forming large accumulations expanding the alveoli	
	Hemosiderophages	None	Scattered hemosiderophages (not forming clusters) are in < 2 10X HPF. Perls usually needed to confirm their presence	Moderate number of hemosiderophages forming clusters in 2-4, 10X HPF. Perls' not needed to observed them	Large clusters of hemosiderophages in 4-5, 10X HPF.	
	Proliferation of type II pneumocytes	None	Scattered individual subpleural alveoli lined by single cordon in < 2, 10X HPFs	Cluster of more than 3 subpleural alveoli lined by discontinuous cordons of type II pneumocytes	Large coalescing foci of supleural alveoli entirely lined by type II pneumocytes in more than 2, 10X HPFs	
	Iron encrustation	None	Isolated collagen bundles display iron encrustation in 1, 10X HPF	Multiple collagen bundles display iron encrustation in 2-3, 10X HPF	Multiple collagen bundles display iron encrustation in >3, 10X HPF	
**Vessels**	Vascular remodeling	None	Rare, detected in 1-2 vessels mainly characterised by mild thickening	Multiple, detected in 3-6 vessels and in 2-3, 10X HPF with evident marked adventitial thickening	Common, in more than 7 vessels and/or in more than 4, 10 X HPF	
	Adventitial iron encrustation	None	Rare, detected in 1-2 vessels in less than 2, 10X HPF	Multiple in 3-6 vesssels and in 2-3, 10X HPF.	Common, in more than 7 vessels and/or in more than 4, 10 X HPF	
	Adventitial Hemosiderophages	None	Scattered hemosiderophages (not forming cluster) in 2 vessels	Moderate hemosiderophages forming rare clusters in 3-6 vessels	Large hemosiderophages clusters in more than 6 vessels	

l.p.- lamina propria

Supplemental table S3: Anamnestic data for each horse.

Horse ID	Group	Sex	Breed	Age (years)	Race type	Humidity (%)	Temperature ºC	Euthanasia	Season	HW/BW ratio
1	Control	Gelding	Thoroughbred	3	N/A	N/A	N/A	yes	Winter	N/A
2	Control	Male	Arab	7	N/A	N/A	N/A	yes	Summer	N/A
3	Control	Female	Thoroughbred	2	Flat	87	10.7	yes	Autumn	0.97
4	Control	Female	Irish Draughtx	17	N/A	N/A	N/A	no	Spring	0.66
5	Control	Male	Welsh	1	N/A	N/A	N/A	no	Spring	N/A
6	EIPH	Female	Thoroughbred	4	Flat	76	14	yes	Summer	0.94
7	EIPH	Gelding	Thoroughbred	8	Jump	73	20.5	no	Autumn	0.87
8	EIPH	Gelding	Thoroughbred	8	Jump	66	10	yes	Autumn	1.04
9	EIPH	Gelding	Thoroughbred	8	Jump	N/A	6.6	no	Autumn	N/A
10	EIPH	Gelding	Thoroughbred	12	Jump	92	10	yes	Spring	1.01
11	EIPH	Female	Thoroughbred	7	Jump	70	7	yes	Spring	1.19
12	EIPH	Gelding	Thoroughbred	8	Jump	47	15	yes	Spring	0.91
13	EIPH	Gelding	Thoroughbred	7	Jump	50	14	yes	Spring	1.00
14	EIPH	Female	Thoroughbred	7	Jump	90	9.1	yes	Winter	0.93
15	EIPH	Gelding	Thoroughbred	6	Jump	98	7.5	yes	Winter	0.94
16	EAFPH	Gelding	Thoroughbred	6	Flat	88	7.7	no	Autumn	0.99
17	EAFPH	Gelding	Thoroughbred	8	Jump	95	1.6	no	Winter	N/A
18	EAFPH	Gelding	Thoroughbred	8	Flat	75	3.9	no	Winter	0.97
19	EAFPH	Female	Thoroughbred	6	Jump	51	14.7	yes	Winter	0.88
20	EAFPH	Gelding	Thoroughbred	4	Jump	49	11	no	Spring	0.89
21	EAFPH	Female	Thoroughbred	9	Jump	89	9	no	Spring	1.00
22	EAFPH	Female	Thoroughbred	8	Jump	55	19	no	Summer	1.10
23	EAFPH	Gelding	Thoroughbred	7	Jump	89	7.6	no	Summer	1.07
24	EAFPH	Gelding	Thoroughbred	3	Flat	78	8.7	no	Winter	0.90
25	EAFPH	Gelding	Thoroughbred	5	Jump	96	8.5	no	Winter	N/A

EIPH: exercise induced pulmonary hemorrhage;
EAFPH: exercise associated fatal pulmonary hemorrhage
HW/BW: heart weight/ body weight

Supplemental table S4: Perivascular collagen D period and fibrillar diameter measures

		Collagen Fibril diameter				Collagen D period length			
Horse	Group	Mean	SD	Min	Max	Mean	SD	Min	Max
1	Control	51.1	7.6	39.8	68.1	55.28	5.039334	43.088	65.032
2	Control	47	4.9	38.8	56.4	49.46	8.878015	41.495	67.455
9	EIPH	43.2	7.5	36.3	70.8	58.50	5.491672	47.232	69.304
10	EIPH	50.65	8.4	38.2	53.3	52.73	4.567636	45.006	63.28
13	EIPH	44.8	4	38.3	53.3	54.55	4.026314	45.497	62.765
16	EAFPH	56.4	11.4	39.9	75.2	53.98	3.911274	44.644	60.343
18	EAFPH	41.7	6.3	31.4	54.3	51.42	6.834564	43.592	65.999
19	EAFPH	56.5	10.6	36.8	76.8	57.18	3.536845	48.779	64.336
